# Why give birth in health facility? Users’ and providers’ accounts of poor quality of birth care in Tanzania

**DOI:** 10.1186/1472-6963-13-174

**Published:** 2013-05-10

**Authors:** Lilian T Mselle, Karen Marie Moland, Abu Mvungi, Bjorg Evjen-Olsen, Thecla W Kohi

**Affiliations:** 1 School of Nursing, Muhimbili University of Health and Allied Sciences, Dar es Salaam, Tanzania; 2 Schools of Nursing, Bergen University College, Bergen, Norway; 3 School of Social Work, Dar es Salaam, Tanzania; 4 Centre for International Health, Bergen, Norway; 5 Department of Obstetrics and Gynaecology, Sorlandet Hospital, Flekkefjord, Norway

**Keywords:** Health facility, Quality of birth care, Obstetric fistula, Women experiences, Tanzania

## Abstract

**Background:**

In Tanzania, half of all pregnant women access a health facility for delivery. The proportion receiving skilled care at birth is even lower. In order to reduce maternal mortality and morbidity, the government has set out to increase health facility deliveries by skilled care. The aim of this study was to describe the weaknesses in the provision of acceptable and adequate quality care through the accounts of women who have suffered obstetric fistula, nurse-midwives at both BEmOC and CEmOC health facilities and local community members.

**Methods:**

Semi-structured interviews involving 16 women affected by obstetric fistula and five nurse-midwives at maternity wards at both BEmOC and CEmOC health facilities, and Focus Group Discussions with husbands and community members were conducted between October 2008 and February 2010 at Comprehensive Community Based Rehabilitation in Tanzania and Temeke hospitals in Dar es Salaam, and Mpwapwa district in Dodoma region.

**Results:**

Health care users and health providers experienced poor quality caring and working environments in the health facilities. Women in labour lacked support, experienced neglect, as well as physical and verbal abuse. Nurse-midwives lacked supportive supervision, supplies and also seemed to lack motivation.

**Conclusions:**

There was a consensus among women who have suffered serious birth injuries and nurse midwives staffing both BEmOC and CEmOC maternity wards that the quality of care offered to women in birth was inadequate. While the birth accounts of women pointed to failure of care, the nurses described a situation of disempowerment. The bad birth care experiences of women undermine the reputation of the health care system, lower community expectations of facility birth, and sustain high rates of home deliveries. The only way to increase the rate of skilled attendance at birth in the current Tanzanian context is to make facility birth a safer alternative than home birth. The findings from this study indicate that there is a long way to go.

## Background

The underutilisation of health care facilities for delivery in Tanzania is a huge health care problem and a major concern for a country striving to attain Millennium Development Goal (MDG) 5 to reduce maternal mortality by 75% by the year 2015. Estimates indicate that around 50% of births in Tanzania occur in health facilities, of which only 51% are attended by skilled personnel [1]. Although the proportion of women giving birth in health facilities is slowly increasing compared to earlier estimates [2], it stands in sharp contrast with antenatal clinic attendances, which is stable and high at 96% [1]. Increasing the proportion of births with skilled attendance is advocated by international agencies as a key factor in reducing maternal and perinatal mortality and morbidity [3]. However, multiple factors prevent many women from seeking birth care in health facilities [4-7]. For instance, the husbands’ approval is important for women to utilise health facility for delivery [8].

Experience in countries with low maternal mortality reveals that access to good quality obstetric care is vital for reducing maternal mortality [9,10]. More than 80% of maternal deaths worldwide that result from both direct and indirect obstetric complications are preventable [11], given that all women have access to the interventions for preventing or treating pregnancy and birth complications.

The WHO, UNICEF and UNFPA identified a package of 8 interventions [9,12,13] to treat obstetric complications, which are known as emergency obstetric care (EmOC). The “basic emergency obstetric care” (BEmOC) health facilities are those capable of providing parenteral- antibiotics, oxytocic drugs and anticonvulsants for pregnancy-induced hypertension. These facilities should be able to perform manual removal of the placenta, removal of retained products of conception, and perform assisted vaginal delivery. The “comprehensive emergency obstetric care” (CEmOC) health facilities are those capable of providing basic emergency obstetric care and also performing caesarean section and blood transfusion.

The health system in Tanzania is organised in a referral pyramid, starting from dispensaries at the bottom and rural health centres (RHCs) that provide BEmOC and treatment of minor conditions. At the district level, there are district hospitals at first referral level where necessary drugs, equipment and skilled staff are available to provide CEmOC. There are also regional hospitals in each region, with the highest levels being national and specialised hospitals [14]. Birth care is provided in all levels of health facilities depending on the birth care need of a woman (i.e. BEmOC or CEmOC). According to the Ministry of Health and Social Welfare, birth care should be offered free at both public and private health facilities at all levels [15].

Worldwide, lack of access to EmOC is one of the major obstacles in attaining MDGs 4 on child health and survival and 5 on maternal health. With an estimated maternal mortality ratio of 454 per 100,000 live births [1], Tanzania is one of the countries in sub Saharan Africa with unacceptably high numbers of maternal deaths. Another outcome of inadequate care at birth is disabling obstetric complications, with more than a quarter million women suffering these in Tanzania alone. The most disabling one is obstetric fistula, which affects between 50,000 to 100,000 of women each year worldwide [16], and between 2500 and 3000 in Tanzania [17].

Drawing upon the availability, accessibility, acceptability and quality of care (AAAQ) framework [18], this study used the concept of acceptability and quality of care to understand the quality of birth care offered to women in health facilities in Tanzania. Quality of care refers to ‘doing the right things right, obtaining the best possible clinical outcome, satisfying all customers, retaining talented staff and maintaining sound financial performance’ [19] whereas, acceptability refers to organisational, ethical and cultural factors that can facilitate or hinder use of services [20].

Studies conducted in Tanzania have pointed out health system factors that prohibit women from seeking birth care in health facilities [5,6,21]. In these studies, informants were antenatal mothers and women during labour and delivery. This paper reports weakness in the provision of acceptable and adequate quality of birth care at health facilities using as an entry point the experiences of women who survived, but experienced serious complications during childbirth. The outcome was perinatal death and obstetric fistula. Further, the experiences of husbands, community peers, and nurse-midwives working in the labour wards at both BEmOC and CEmOC health referral system where the women were referred during labour were included in the study.

## Methods

### Study design and setting

The qualitative research was carried out between August 2008 and August 2010, at Comprehensive Community Based Rehabilitation in Tanzania (CCBRT) [5], Temeke district hospital in Dar es Salaam, and Mpwapwa district in Dodoma region. Temeke hospital operates as a municipal and district hospital in Dar es Salaam city, and has 26,568 annual numbers of deliveries, calculated out of total annual expected deliveries [22]. The CCBRT hospital was chosen because it was among the two major service points for fistula surgery, with higher fistula repair rates [23]. Mpwapwa district on the other hand, was chosen because many women affected by obstetric fistula who were admitted at CCBRT during the study period were from this district. Selection of these settings ensured access to an adequate number of women affected by fistula.

### Informants and data collection

The focus of this study was experiences related to obstetric fistula. Therefore 16 women affected by obstetric fistula (see Table 1) were conveniently selected from CCBRT hospital. The inclusion criteria were that the woman was admitted at the hospital for obstetric fistula repair before or after surgery, was able to speak Swahili, and agreed to participate in the study. A senior nurse-midwife (not part of the interviewed nurse- midwives) identified women affected by obstetric fistula at CCBRT during the data collection period. The purpose of the study and principles of confidentiality were explained, and thereafter, a convenient time for an interview was arranged. This study also involved six husbands of the affected women, six community members and five nurse-midwives staffing labour wards at both BEmOC and CEmOC health facilities. Community members were purposefully selected from different locations in Mpwapwa district, based on gender and occupation, whereby husbands and nurse-midwives were conveniently selected. Husbands and community members were recruited from Mpwapwa district in Dodoma region, with assistance of the Anti-Female Genital Mutilation Network project (AFNET) [24], whereas nurse-midwives were selected from Mboli dispensary and, Mpwapwa, and Temeke district hospitals.

**Table 1:** Characteristics of women affected by obstetric fistula who were interviewed at CCBRT hospital, Tanzania, 2008–2010

**Woman**	**Age (years)**	**Marital status**	**Years in school**	**Duration with Fistula**	**Domicile**
1	19	Single	7	4 months	Morogoro
2	25	Married	3	>1 year	Tanga
3	35	Single	7	18 years	Kilwa
4	20	Divorced	7	6 months	Pwani
5	28	Married	12	2 months	Dar es Salaam
6	30	Married	12	3 months	Iringa
7	33	Divorced	7	19 years	Morogoro
8	29	Divorced	0	10 years	Morogoro
9	35	Married	7	2 years	Singida
10	40	Married	7	1 year	Dodoma
11	43	Separated	7	20 years	Dodoma
12	40	Single	7	20 years	Singida
13	25	Divorced	7	8 months	Iringa
14	28	Divorced	7	12 years	Dodoma
15	29	Divorced	0	9 years	Dodoma
16	18	Single	0	3 months	Dodoma

#### Semi-structured interviews

Semi structured interviews [25] with women affected by obstetric fistula, which lasted between 45 minutes and 2 hours, were conducted in person by the first author (LTM), a registered nurse with a background in social sciences and health promotion, and a fluent Swahili speaker. The sample size of the women affected by obstetric fistula was not predetermined. However, saturation [26] was achieved after 16 interviews, where answers from women seemed to repeat information gained earlier and little new information was attained. Interviews were done at CCBRT hospital, in a private room adjacent to the fistula ward.

Five individual face-to-face interviews were conducted with nurse-midwives who were on duty during the data collection period. Of these, three were from the district hospital in Dar es Salaam, two from Mpwapwa district; one from a rural dispensary and another from the district hospital. It was important to interview nurse-midwives employed at different levels of health care, since the women who had suffered obstetric fistula were referred from lower (BEmOC) to higher (CEmOC) levels of care during labour. The interview with nurse-midwives lasted between 30 and 40 minutes and were held in their respective health facilities, after duty, when they were about to go home. The interview guide had only one question with few probing questions focusing on the nurse- midwives’ experiences of providing birth care.

Interview guides with open-ended questions and probes used were not rigidly adhered to, allowing the interviewer to explore issues as they emerged [27], and they were revised during the course of data collection. All interviews were audio-recorded with permission from informants. Notation of nonverbal expressions of the informants during the interview was taken during and immediately after the interview.

#### Focus group discussion

Two FGDs each with 6 participants were held at Mpwapwa district in Dodoma region by the first author using the FGD guides. The FGD with community members was conducted at the AFNET office, Mpwapwa branch. The discussion begun by reading aloud a hypothetical scenario describing potential challenges women face in relation to seeking adequate birth care. This was done to stimulate and guide the discussions. The group included a teacher, a farmer, a homemaker, a volunteer at AFNET, an accountant and a traditional birth attendant who had also suffered obstetric fistula.

The other FGD with husbands of women affected by fistula was held at Tambi village. Ten husbands were approached and all agreed to take part in the study, however, only six husbands who came from different places of Mpwapwa district turned up on the day of discussion. The group was comprised of husbands of different ages and ethnicity. Discussions were conducted in Swahili, lasted for 1–2 hours and were audio-recorded.

### Data analysis

Analysis of the interviews was done concurrently with the data collection process. All interviews and FGDs were transcribed verbatim, and translated from Swahili to English by a linguistic teacher with the first author doing the final editing and ensuring accurate translation. The English transcripts were used for analysis, and the original transcripts were crosschecked to ensure a correct interpretation throughout the process. The analysis was guided by a thematic analysis approach [28]. All transcripts were carefully read sentence by sentence to obtain a sense of the content as narrated by informants. Phrases and sentences related to experiences and perspectives related to institutional maternal care were coded in the margin of the transcript sheets by the first author, and codes with similar content were brought together into themes (see example, Table 2). Several rounds of coding and discussions among the co-authors were necessary for content validity [29]. To ensure confirmability, all the co-authors reflected, discussed differences in interpretation of data, and agreed on the categorisation [30].

**Table 2:** An initial attempt to codify “missing attention, care, and support” theme from accounts of health care users, by means of inductive thematic

**Codes**	**Themes**	**Relevant quotes**
Pushed without instruction	Missing attention, care, and support	I arrived at the hospital at midnight, but the way I was received was not good. When I walked into the hospital, the labour pains started getting stronger, but nurses did not receive me well.… They told me I should wait for the doctor to come. (Woman 6)
Bad reception		…you find a woman has been in labour for a long time, but they are relaxed in their offices, providing no assistance. (Husbands’ FGD)
Asked to wait		
No assistance		
Did not check anything on me		There [at the health facility], a woman in labour was calling for help. Nurses were sitting in the office; the woman’s mother and I were sitting outside. Her mother was complaining to the nurse....Why can’t you help my daughter who has been crying for help for a long time? They responded by saying behind the closed door, “Go and help her yourself.” (Community members’ FGD)
Sitting in the office chatting		
No care		
I was alone throughout labour		
No help		
		I was alone and nurses were there but they did not help me..... (Woman 6)
No examination		
		I stayed for a long time, and each time I called for help the nurse would tell me to lie on my side. (Woman 7)
		A young woman pushed without any instructions; she called the midwives for help [but] they did not pay attention. Then the young woman got tired, [and] the baby died in the womb. (Community members’ FGD)
Left alone throughout the night		… they did nothing; they did not check anything on me. I was alone throughout the night; there was no doctor. (Woman 3)
		I was not assisted in any way; I just pushed on my own. (Woman 7)
		They did not do any examination until the third day, when the doctor came in the afternoon. Thereafter, the doctor told me that the foetus had died in the womb. (Woman 1)
		I was alone throughout the night; there was no doctor. (Woman 3)
Stayed for long time		
Crying for help for a long time		
Did nothing on me		
They did nothing		
Pushed on my own did not pay attention		
Push without instruction		
Not assisted in any way		
No doctor		

### Ethical consideration

Ethical clearance was obtained from the Muhimbili University of Health and Allied Health Sciences (MUHAS) Research and Ethical Review Board. Permission to conduct the study was thereafter obtained from CCBRT hospital and the Municipal director, Temeke municipality; Dar es Salaam. All informants provided written consent after discussing the purpose of the study and issues of confidentiality.

## Results

Health care users and providers expressed great dissatisfaction with the care and working environment at health facilities. In addition health care users lacked trust in the health facility while the nurse-midwives felt generally disempowered. As shown in Table 3, three categories emerged from the experiences and views of women, husbands’ and community members (users) on maternity care, and two categories of experiences of working in the labour wards emerged from nurse-midwives’ (providers). Expressions in Swahili are given with quotation marks and fictitious names are used in the quotes.

**Table 3:** Users and providers experiences on maternal care

**Users’ experiences**	**Providers’ experiences**
1. Missing necessary services	1. Lacking supportive supervision and supplies
2. Missing attention, support and care	2. Lacking motivation to work effectively
3. Experiencing physical and verbal abuse	

### User perspectives

#### Missing necessary services

Health facilities are frequently far away from where many women live and women have to walk for many hours, or spend money on transport to get to them [5]. Women reported that sometimes when they go to the health facilities for check-ups, they do not find health providers to attend to them, and if available, they do not attend to their needs to their satisfaction:

*(…) as you know the set up in village health facilities; sometimes when you go there you do not find them (health providers), and if you do find them they do not care or check you properly, neither tell you about your progress* (Woman 11).

Women also reported that in health facilities, their progress of labour was not continuously monitored since they were left alone most of the time during labour. According to the interviewed women, nurses did not assess them during this crucial and critical period where their lives and that of their babies were at stake:

*I was alone and nurses were there but they did not help me (…). The following morning at 7am while in the labour ward, I heard the voice of my neighbour at home, I asked her to come in. When she saw the state I was in, she called the doctor, and that is when the services started, until operation* (Woman 6)*.*

Some of them had to give birth by themselves without support, as one woman explained during the interview: “*I was not assisted in any way; I just pushed by myself”* (Woman 7). Similarly, community members pointed out the issue of staff shortage and underscored the need for the trained traditional birth attendants (TBAs) to be invited to team up with midwives to provide birth care:

*(…) there are traditional birth attendants (TBAs) who have been trained, why are they not helping in dispensaries? Particularly, when there are many women in labour? These women have been trained, they have acquired skills. They could team up with nurses to help women during labour. They could do just the way they do with elders (jurors) in courts. These TBAs could be given allowances, while nurses keep their salaries; can’t you see that these TBAs will save women’s lives?* (A female participant in the community FGD).

Availability of safe blood in the CEmOC facilities is a key component of emergency obstetric care [31]. Women interviewed reported that there was no blood readily available for transfusion in the CEmOC health facilities and therefore if a woman was in need of blood transfusion, relatives had to donate blood or pay to get blood, regardless of the woman’s condition:

*(…) When I came back from the theatre, I was told that I had severe anaemia and malaria…my father donated one unit of blood and had to sell his bicycle so that we could get cash to pay for two more units of blood* (Woman *16).*

#### Missing attention, support and care

Positive clinical interaction is influenced by a sense of kindness, understanding and active listening [32]. According to narratives from informants, these qualities were generally missing in this study. Women were not happy with the attitude of the nurse-midwives and felt neglected when they failed to receive attention and support from them. One woman, who reached the hospital immediately after onset of labour, described the unfriendly reception by nurses working in the labour ward:

*I arrived at the hospital at midnight but the way I was received was not good. When I walked into the hospital, the labour pains started getting stronger, but nurses did not receive me well. When I showed them my clinic card, they complained that I was living nearby (Iringa road) and why did I come to hospital at midnight. They said this was the behaviour of women from town to delay to come to hospital because they know the hospital is near. They told me I should wait for the doctor to come because they had already seen my clinic card* (Woman 6).

Husbands who had accompanied their wives to the health facility at the time of delivery also echoed women’s experience of lack of support from nurse-midwives during labour and delivery:

*(…) my wife started labour at 2am, we arrived in hospital at 9am. However, until 3pm, we saw nobody. Could you really say that this ward has health care providers? (…) From 9am until 3pm. (…) by our own effort, we traced the doctor to his house; there we had to beg…while getting abusive language (…). We asked the doctor to tell us if there was no means of helping our patient, she should be discharged, so that we leave early, since it would be best to let her go and die at home rather than die in the facility because her transportation will be very costly* (Husbands’ FGD).

They voiced serious criticism on the indifference they had experienced from the nurses and blamed them for the negative birth outcomes:

*Midwives contribute to the occurrence of fistulas (…) you find a woman has been in labour for a long time, but they are relaxed in their offices, providing no assistance* (Husbands’ FGD).

The community members shared the concern shown by husbands on the inadequacy of the care provided by nurses. One community member during discussion gave vivid experience of poor quality services:

*There (at the health facility), a woman in labour was calling for help. Nurses were sitting in the office; the woman’s mother and I were sitting outside, her mother was complaining to the nurse…why can’t you help my daughter who is crying for help for a long time? They responded by saying behind the closed door “go and help her yourself’. By then the baby was stuck in the pelvis, could not come out and eventually the baby died right there* (Female participant -FGD community members).

However, another member during discussion gave a different opinion that nurses work for long hours without being adequately compensated and therefore they need to be encouraged to work effectively:

*Nurses need to be motivated, because they work under very difficult conditions. They do not do overtime which could help them earn extra money; hence, they are less motivated to work. (…) they work for long hours and in a shift, sometimes a nurse is not relieved from duty because another nurse has a problem and did not come on time. But given the nature of the job, the nurse cannot just leave patients behind, although she could be really tired and has to continue working. She otherwise could have gone home and do other income generating activities, but she cannot (…). Nurses are paid less, no incentives/allowances, no payment for extra work done, the government should really look at this* (Male participant-FGD community members).

#### Experiencing physical and verbal abuse

Physical abuse was part of the poor treatment women received during labour. This was evident from the community members’ discussion where one member explained:

*A young woman pushes without any instructions, she calls the midwives for help, they do not pay attention, we were there. Then the young woman got tired, the baby dies in the womb (…). When they approached the woman, they started asking why she did not push, after which they slapped her. I had witnessed this happening to my niece, the baby was well, the mother started calling nurses to help, they never responded, they were just sitting there; later on they started beating her, instead of helping her, until the baby died, we were outside listening to all that was happening* (Female participant -FGD community members)*.*

Husbands did not have good things to say about midwives. Their negative experiences with these health personnel led them to question whether the nurses who provide care to women during labour had proper professional training, and if so, why don’t they put it into practice? This was well illustrated by a husband who reported his experience:

*I am afraid, some of the midwives do not practice what they were taught, and should that be the case, then, perhaps they did not get adequate training. Because whereas women are crying in pain asking for help (…) what the midwife does… does not show that she is really nursing patients… she tells the patient “… you will see, when you were making your baby I was not with you (…), keep on crying, when you die we will come to collect your dead body....” I said to myself that this is tragic* (Husbands’ FGD).

Husbands were not alone in doubting the professional competence of the nurse-midwives. Discussion with community members showed that they also questioned the skills of nurse-midwives. As one member narrated:

*(…) nurses’ actions were very unprofessional, if they adhered to what they have been trained to do, they could have been very helpful, such that when the doctor comes, he attends those with complications. Without such action, women will continue to die or get fistula in health care facilities* (Male participant - FGD community members)*.*

### Provider perspectives

#### Lacking supportive supervision and supplies

This study revealed that nurse-midwives working in the labour wards lacked supportive supervision and supplies that are critical in executing their duties. They reflected on the lack of materials, drugs and safe blood for transfusions in the health facilities that are basic and essential tools to ensure that they can carry out their duties effectively. The situation is expected to be different in the health facilities providing CEmOC, in that at these facilities blood transfusion should always be readily available for emergency cases [33]. However, in this study it was revealed that even in district hospitals the availability of blood for transfusion was a serious problem. A midwife working at a district hospital located in Dar es Salaam city indicated that it was a common practice to refer women in labour to the National Hospital for blood transfusion:

*If a woman has low haemoglobin level and requires blood transfusion (…), she will be referred to Muhimbili National Hospital* (Midwife from Temeke, Dar es Salaam).

Nurse-midwives also do not get sufficient supplies, which deters their desire to help women during labour and delivery. As the following quotation illustrates:

*(…) the gloves we are given are not enough, so we send the message to the clinics that when women come to deliver, they should bring along cotton wool and gloves. Because if they come and there are no gloves they may not be attended* (Nurse-midwife from Temeke, Dar es Salaam).

As a result of lack of equipment and severe shortage of supplies necessary for delivery in the health facilities, nurse-midwives see themselves as working in very difficult environments that hinders their ability to provide quality birth care. A nurse-midwife working in the district health facility expressed the poor state of affairs in the following words:

*(…) listen, at this facility, things like gloves we are given only one box. Right now as you can see, there are not as many women in labour as it used to be (…). There are times when during a night shift you could deliver 40 or more babies alone (…) the gloves we are given are not enough,(…) you need to protect yourself, if you put on only one pair of glove, some blood could seep into your fingers. Therefore, if women come at least with gloves and cotton wool, even when the hospital runs out of supplies, we could still help* (Nurse-midwife from Temeke, Dar es Salaam).

An increased proportion of births attended by skilled attendants is a significant factor in reducing maternal and perinatal mortality and morbidity. However, in this study shortage of skilled attendants in health facilities was found to be critical. Nurse-midwives reported being over-burdened by the number of women coming for deliveries due to shortages of personnel. To deal with this, they had to work overtime, select whom they should give care to (serious cases), and leave other women to give birth on their own without support, as highlighted below:

*(…) sometimes while you are assisting one woman to deliver, you could find the other woman has delivered on her own, you may find the head is completely out. What helps is that we put on three or four pairs of gloves at once, in an effort to take care of such cases, because you may be helping one woman and you oversee another one pushing the baby, so what you do is you tie the cord of the baby and give the baby to her mom and you would remove a pair of glove and fast you go help that woman* (Nurse-midwife from Temeke Dar es Salaam).

Another nurse-midwife shared:

*Because of the shortage, I only assess those who are seriously in need of immediate attention, and we have a signboard that we place to identify serious cases, which need close attention* (Nurse-midwife from Temeke Dar es Salaam).

When asked what she meant by serious cases she responded:

*A mother who is hypertensive, one with a previous scar, first pregnancy, short stature, and a woman who delivered many times (multipara*) (Nurse-midwife from Temeke, Dar es Salaam).

Another midwife working in a low-level health facility in Mpwapwa district shared her experience of being exhausted from being on duty for a long time taking care of women in labour:

*There is critical shortage of staff, for example in our dispensary, we are only two, a clinical officer and I, and we have delivery services. Therefore, sometimes we are forced to stay overnight to help women in labour. Beside I am also supposed to take care of all other units in the dispensary such as injection, antenatal care, children, and dressings. It is not easy* (Nurse-midwife from Mboli village, Mpwapwa).

Nurse-midwives often had to assist more than one woman at the same time and this compromised the quality of care provided. They were commonly providing rushed care or left women by themselves half way through labour to take care of another woman who was in critical need of help:

*(…) Sometimes the ward is flooded, with some mothers sleeping on the floor. Therefore, you need to be ready and prepared all the time. You could be supporting one woman to deliver, while at the same time you see another woman pushing. Therefore, what you do is to tie the cord of the baby you have just delivered, quickly change gloves and fast rush to the next woman to assist her deliver…and the cycle continues* (Nurse-midwife from Temeke Dar es Salaam)*.*

Nurse-midwives explained that they were forced to act the way they did to salvage lives. For example, when asked as to why they sometimes abuse women during labour, they claimed that they did so to ensure women and their babies come out of labour safely:

*You know, sometimes you need to slap them as a way of forcing them to push the baby! You know in our hospital if you deliver a stillbirth or you get a maternal death …mmh is “kasheshe” (you will be in big trouble) you will be required by administrators to write a statement of what happened and you could even lose a job. Therefore, to avoid all this you need to try to assist women give birth safely* (Nurse-midwife from Mpwapwa district).

A stillbirth or a death of the mother during childbirth may be accompanied by serious sanctions and was greatly feared as explained by a nurse-midwife from Temeke district hospital:

(…) besides if you get a maternal death you would wish the land to part and bury yourself. Therefore, you really have to make sure that during your shift you do not get maternal death or stillbirth.

#### Lacking motivation to work effectively

Lack of motivation was a major concern among nurse-midwives who were interviewed. They claimed that although they work in poor and unsupportive environments that are overcrowded, with limited equipment, supplies and personnel, their work was never appreciated by their supervisors:

*(…) you know, it is very important to show appreciation and encourage people…even by just calling them and acknowledging the work they do, it gives courage and it helps (…)* (Nurse-midwife from Temeke Dar es Salaam).

Another midwife said:

*Truly, we are working hard and in a difficult situation (…), at least we could have been motivated, you understand (…). For example after a night shift, in the morning when you meet your ‘in charge’, if she could simply say, “ooh you have done a good job”, it encourages, and makes you feel that your work is being appreciated* (Nurse-midwife from Temeke, Dar es Salaam).

Probably because of overcrowding and overwork, lack of supplies and blood exposure, working in the labour ward was regarded as a punishment from the superiors. This resulted in demoralisation of those assigned to work there. A nurse-midwife working in the labour ward reported in an interview that a nurse could be transferred to work in the labour ward, not because she has the required competence, but rather as a punishment because she does not have a good relationship with her supervisor. This practice demoralised nurse-midwives. The labour ward is perceived to be a relatively busy ward compared with other wards.

## Discussion

This paper has explored weaknesses in the provision of birth care as experienced by women who acquired serious birth injuries due to sub-optimal birth care, their husbands and community members on the one hand and nurse-midwives working at both BEmOC and CEmOC health facilities on the other. The paper describes challenges related to relational as well as structural issues of care. The findings point to problems of acceptability and of the quality of maternal care. The lack of respect for women’s dignity and rights to safe delivery is alarming.

### Study limitations

The selection of women was restricted to those who had a bad obstetric history and developed obstetric fistula as an outcome of labour. In tracing weaknesses in health care delivery, narratives from women who had normal deliveries may have been a useful comparison. Hospital data, such as availability of equipment, drugs and supplies and the presence of skilled personnel, which are known determinants of the quality of care, were not collected. This would have helped in examining the information provided by informants on the inadequate situation prevailing in the health facilities. Some women in this study had lived with fistula for many years and may not have accurately remembered the events that led to a birth that occurred many years ago. There is a possibility of over-reporting their negative experiences of birth care, and that they could have assimilated the experience of others into their own. Nevertheless, their openness and their instant and consistent responses to the questions and probes during interviews left little doubt that these women recalled their birth experiences accurately. Research indicates that women remember the birthing event and feelings surrounding labour and delivery for many years even when the process is uncomplicated [34].

### Dwindling trust in the health facilities

Inadequate resources and lack of skilled attendants are the most often cited reasons for poor quality of birth care. This study revealed that women were assisted during delivery by nurse-midwives working single-handedly, whilst some gave birth without assistance, others received incomplete or rushed birth care or were left half way through delivery. Leaving a woman alone during delivery is dangerous, because complications requiring EmOC are frequently unpredictable and prompt care is crucial [35,36]. For example, delay in detecting post-partum haemorrhage can lead to maternal death. Post-partum haemorrhage alone is responsible for 31% of all maternal deaths occurring in sub-Saharan Africa [37]. The immediate and skilled care during labour and delivery is crucial for safety of the future mothers and their new-borns, because life-threatening complications are largely unpredictable, and become life threatening within a short period.

Researchers [38] have reported that shortage of staff reduces quality of care with increasing workloads making infection control more difficult. The phenomenon of a midwife assisting more than one woman at a time during delivery is a recipe for cross contamination and spread of blood-borne infections between women in labour. Some nurse-midwives in this study appeared to lack motivation due to lack of staff to give a helping hand or support, absence of essential working tools including basic supplies such as gloves, and lack of recognition and encouragement by supervisors.

Staff motivation [39] and solid health systems are linked with provision of better quality services [40]. This may result in women’s improved experience, trust and confidence in the caregivers, which in itself is a determinant for positive pregnancy outcome [41], and thus a source of encouragement for women to seek care in the facility. It is also acknowledged that the way women perceive access to and quality of care is closely linked to their health seeking behaviour [42]. The Tanzanian Government through Primary Health Services Development Programme (PHSDP) 2007–2017, has the intention to address the unsatisfactory performance of the health sector by a rapid increase in the number of health care workers to fill the existing staffing gaps within the health sector [43]. While focusing on increasing the number of health care workers, there is also a need to ensure that nurse-midwives have the necessary competence to work in the labour ward, and are encouraged through supportive supervision and acknowledgement of their work. Further, it is important to equip health facilities with adequate drugs, supplies and equipment for safe delivery.

### Disempowerment of nurse-midwives

Overall, the poor quality of maternal care is the cumulative result of deficiencies in health facilities. This study indicates that health facilities lacked equipment, supplies, drugs and blood for transfusion required during pregnancy and delivery, which is consistent with observations made by others [44,45]. Because of the inadequacies in health facilities, women in this study received poor quality of birth care. For example, many women reported missing drugs, blood for transfusion and other supplies pertinent for pregnancy and delivery in the health care facilities, forcing them to purchase the same from elsewhere. Similar findings were found by others [44], where the families of women in labour were asked to buy drugs, cotton wool and blood for transfusion in health facilities where these necessities were supposed to be available.

In Tanzania, as in most sub Saharan African countries, there is a severe shortage of health professionals across all cadres related to labour and delivery [46]. Shortage of midwives was also a critical problem noted in this study. This is consistent with another study undertaken in Tanzania, that indicated a very low availability of health care staff [6], which in turn may affect the quality of birth care offered. There are currently only 35,202 health care providers, whereas the current number of health facilities requires 125,824 health workers, indicating a gap of 90,722 health workers [47]. This represents a 65% healthcare staff shortage in the public sector and 86% in the private sector. Therefore, the few nurse-midwives available are overwhelmed by women in labour wards, needing their support and care. Availability of skilled health personnel is pivotal to ensuring provision of high-quality health care [48], and the lack of skilled personnel contributes to poor maternal outcomes.

Due to the inadequate service provided by nurse-midwives, community members suggested that TBAs should be invited to work alongside nurse-midwives in the health facilities as a means of reducing the burden on the few nurse-midwives available. However, the Tanzanian government no longer recognises TBAs as partners in maternal health and has stopped training them [49]. Previous studies have shown the usefulness of TBAs as a strategy to reduce delays that occur at home [50], but there is no evidence that TBAs will reduce delays or improve the quality of care within the health facilities.

The Government of Tanzanian through the Ministry of Health and Social Welfare (MoHSW) is responsible for ensuring that health personnel, equipment and drugs are available at all levels of health facility. However, because of the progressive reduction of the Government’s budgetary allocation to health every year, it is perhaps not surprising to see the continued deterioration of care at health facilities. For example, in the fiscal year 2011/12, the budgetary allocation of 8.9% for health [51], was below the 15% recommended by the Abuja declaration in 2000, as a minimum budget for health to improve quality of care [52], indicating that the government needs to increase health budget allocation in order to improve care.

### Nurse-midwives and the experience of moral distress

Inadequate healthcare facilities are not the only factor associated with poor provision of maternal care, poor attitudes of nurse-midwives is another major factor. In line with other studies [5,53], some of the women in this study received an unwelcome reception during admission onto the labour ward. Some of them were left to give birth alone without assistance. Some revealed that when they shouted for help, the nurse-midwives did nothing. In the few cases, when nurse-midwives came at all, some of the women experienced verbal and physical abuse. Such improper conduct by nurse-midwives during the provision of care to women during labour and delivery breached the nursing code of ethical conduct, grounded in the philosophical ethical principle of fidelity and respect for dignity, worth, and self-determination of persons [54].

The nurse-midwives’ inappropriate attitude toward women in labour may be caused by nurse-midwives experience of moral distress. Moral distress [55-57] evidenced by negative feelings, powerlessness, conflicting loyalties and uncertainty threatens the delivery of good patient care. It may arise when one knows the right thing to do, but personal or institutional constraints make it impossible to pursue the right course of action [57,58]. Lack of nurse-midwives motivation in terms of low pay, long working hours and working in dilapidated, overcrowded and inadequate health facilities, with no training opportunities, together with lack of support from their superiors may have affected their conduct. This could partly explain why even in relatively well-equipped health facilities, women were still humiliated by midwives. As shown by Jewkes and colleagues [53], frequent and often violent abuse of patients can be caused by a complex interplay of concerns, including organisational issues, professional insecurities, a perceived need to assert control over the environment and sanctioning of the use of coercive and punitive measures to do so.

## Conclusions

The poor quality of birth care experienced by women affected by obstetric fistula seem to be attributed to inadequacies in health facilities, including lack of adequate numbers of nurse-midwives, equipment, supplies, drugs and blood for transfusion during pregnancy and delivery. The Government through MoHSW must improve the working environment in health facilities in order to encourage women to utilise these facilities and motivate nurse-midwives to provide better birth care. The Government must ensure that health facilities are well equipped, guidelines are provided, and that these are followed. Instead of using absence of maternal death as a positive indicator of maternal care, close monitoring and supervision of the process of birth care provision should also be considered. Furthermore, health care facilities need motivated, competent and adequate numbers of nurse-midwives equipped with the essential supplies and drugs, supported by a policy and regulatory framework for them to function effectively. Overall, lack or poor provision of birth care causes women to lose trust in health care facilities, pushing them away from seeking skilled attendance at delivery. Thus, the achievement of the Millennium Development Goals of reducing neonatal and maternal deaths and disabilities may be seriously hampered.

## Competing interests

The author(s) declare that they have no competing interests.

## Authors’ contributions

LTM conceptualised, designed, organised and collected data. LTM and TWK analysed and interpreted the data. LTM drafted the manuscript, which was then critically reviewed and revised by TWK, KMM, BEO and AM. All authors read and approved the final manuscript.

## Pre-publication history

The pre-publication history for this paper can be accessed here:

http://www.biomedcentral.com/1472-6963/13/174/prepub
